# The Effects of Targeted Mild Hypercapnia on Right Ventricular Function After Out-of-Hospital Cardiac Arrest: A Substudy of the Targeted Therapeutic Mild Hypercapnia After Resuscitated Cardiac Arrest Trial

**DOI:** 10.1016/j.chest.2025.11.017

**Published:** 2025-11-24

**Authors:** Mathias Baumann Melberg, Susanne D. Rootwelt, Arnljot Flaa, Geir Øystein Andersen, Kjetil Sunde, Glenn Eastwood, Rinaldo Bellomo, Theresa M. Olasveengen, Eirik Qvigstad

**Affiliations:** a Division of Emergencies and Critical Care, Department of Research and Development, Oslo, VIC, Norway; b Division of Medicine, Department of Cardiology, Oslo, VIC, Norway; c Division of Emergencies and Critical Care, Department of Anaesthesiology and Intensive Care, Oslo University Hospital, Oslo, VIC, Norway; d Institute of Clinical Medicine, University of Oslo, Oslo, VIC, Norway; e Department of Intensive Care, Austin Hospital, Heidelberg, VIC, Australia; f Australian and New Zealand Intensive Care Research Centre, Monash University, Melbourne, VIC, Australia

**Keywords:** hypercapnia, low tidal volume ventilation, out-of-hospital cardiac arrest, right ventricular failure, right ventricular function, TAME cardiac arrest trial

## Abstract

**Background:**

Targeting hypercapnia during invasive mechanical ventilation with subsequent respiratory acidosis may impair right ventricular (RV) function and cause RV failure. RV dysfunction is common after cardiac arrest and may be associated with poor outcomes.

**Research Question:**

Does targeting mild hypercapnia after out-of-hospital cardiac arrest (OHCA) adversely affect RV function, and is RV failure after OHCA associated with increased mortality?

**Study Design and Methods:**

In this single-center, preplanned substudy of the Targeted Therapeutic Mild Hypercapnia After Resuscitated Cardiac Arrest (TAME) trial, patients were randomized to mild hypercapnia (Paco_2_, 50-55 mm Hg) or normocapnia (Paco_2_, 35-45 mm Hg) for 24 hours. Transthoracic echocardiography was performed shortly after ICU admission, during and after the intervention period. Time-matched right heart catheterization (RHC) was performed when available. RV systolic function was assessed with tricuspid annular plane systolic excursion (TAPSE), S′, and fractional area change (FAC), with 2 of 3 measurements less than abnormal thresholds defined as dysfunction. RV failure was defined as RV systolic dysfunction combined with a cardiac index of < 2.0 L/min/m^2^ and central venous pressure of > 12 mm Hg.

**Results:**

A total of 111 patients were randomized and evaluated with echocardiography. RHC was performed in 84 patients. TAPSE similarly was reduced in both treatment groups at ICU admission. During the intervention, TAPSE, S′, and FAC were higher in the hypercapnia group (*P* < .05). Accordingly, 13 patients (25%) in the hypercapnia group demonstrated RV systolic dysfunction compared with 37 patients (64%) in the normocapnia group (*P* < .001). RV failure was present in 30 patients: 8 patients in the hypercapnia group and 22 patients in the normocapnia group (*P* = .011). RV failure was associated with increased 6-month mortality (hazard ratio, 2.89; 95% CI, 1.37-6.10; *P* = .005).

**Interpretation:**

Among patients resuscitated from OHCA, targeting mild hypercapnia compared with normocapnia was not associated with worsened RV function, but rather with less RV dysfunction and failure. RV failure was associated with increased 6-month mortality.

**Clinical Trial Registration:**

ClinicalTrials.gov; No.: NCT03114033; URL: www.clinicaltrials.gov

## Graphical Abstract

**Figure:**
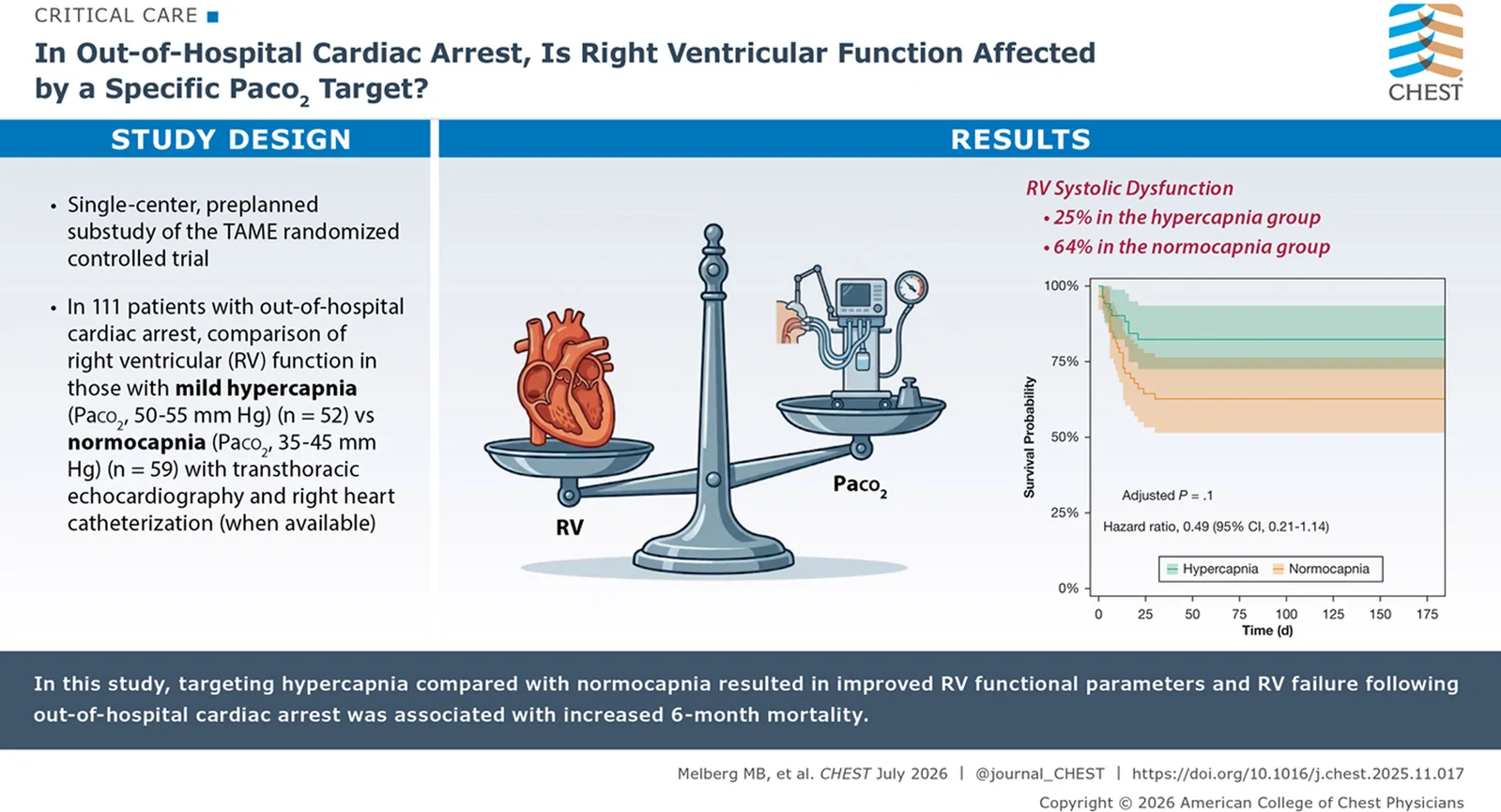

FOR EDITORIAL COMMENT, SEE PAGE 13

**Take-Home Points**

**Research** **Question:** Does targeting mild hypercapnia after out-of-hospital cardiac arrest (OHCA) adversely affect right ventricular (RV) function and is RV failure after OHCA associated with increased mortality?

**Results:** Targeting mild hypercapnia after OHCA was associated with improved RV function, and RV failure was associated with increased mortality at 6 months.

**Interpretation:** Results from this study are contrary to the prevailing view that mild hypercapnic acidosis and low tidal volumes adversely affect RV function and highlight the importance of diagnosing and treating RV failure after cardiac arrest.

Targeting mild hypercapnia (Paco_2_, 50-55 mm Hg) for 24 hours, compared with normocapnia (Paco_2_, 35-45 mm Hg), in the Targeted Therapeutic Mild Hypercapnia After Resuscitated Cardiac Arrest (TAME) trial did not affect neurologic outcomes in patients resuscitated from out-of-hospital cardiac arrest (OHCA).^1^ Although hypercapnic acidosis may increase cerebral perfusion, it can have harmful cardiovascular effects, including reduced myocardial contractility and pulmonary vasoconstriction.^2^, ^3^, ^4^ Hemodynamic instability and increased pulmonary vascular resistance after OHCA has been associated with increased mortality.^5^^,^^6^

Targeting hypercapnia during invasive mechanical ventilation requires adjustments in tidal volume, airway pressures, and respiratory rate, which may impact right ventricular (RV) performance, coronary artery perfusion, and loading conditions.^1^^,^^7^ RV myocardial dysfunction is common after cardiac arrest as part of post-cardiac arrest syndrome, and RV failure has been associated with worse outcomes after cardiac arrest in retrospective studies.^5^^,^^8^, ^9^, ^10^, ^11^, ^12^, ^13^, ^14^ However, prospective data on RV failure and mortality after OHCA are lacking.

Given concerns about inducing hemodynamic instability with hypercapnic acidosis during the first 24 hours of care after resuscitation, we have previously demonstrated that targeting mild hypercapnia, compared with normocapnia, was not associated with increased pulmonary vascular resistance index (PVRI) when assessed by right heart catheterization (RHC).^15^ Conversely, mild hypercapnia was associated with higher mixed venous oxygen saturation, stroke volumes, and cardiac index, possibly because of reduced systemic vascular resistance.^2^^,^^15^

Building on these findings and recognizing the complex nature of RV failure after cardiac arrest, we investigated RV function in patients resuscitated from OHCA. The primary objective was to determine, using serial echocardiography and RHC, whether targeting mild hypercapnia worsened RV function. The secondary objective was to investigate whether RV failure was associated with increased mortality at 6 months.

## Study Design and Methods

### Trial Design and Ethics

This study was a preplanned, prospective substudy of the TAME trial at Oslo University Hospital Ullevål. The TAME trial was an international, investigator-initiated, open-label trial randomizing patients in a 1:1 ratio to mild hypercapnia (Paco_2_, 50-55 mm Hg) or normocapnia (Paco_2_, 35-45 mm Hg) for 24 hours beginning at randomization. Randomization was carried out using random permuted block sizes and was stratified according to trial site. The TAME trial and the present substudy were approved by the regional ethics committee (Identifier: REK 2018/386; ClinicalTrials.gov Identifier: NCT03114033). Patients initially were included and randomized without consent. Written informed consent was obtained from patients’ next of kin in all cases and from patients regaining consciousness after cardiac arrest.

### Patients

Hospitalized comatose adults (18 years of age or older) resuscitated from OHCA of a presumed cardiac cause with sustained return of spontaneous circulation for 20 minutes were eligible for enrolment. Hemodynamically unstable patients and those deemed to be in cardiogenic shock also were included. Complete eligibility criteria are detailed in the main trial publication.^1^ Patients without echocardiographic assessment were excluded from the present study.

### Treatment Protocol

All patients were sedated, intubated, and mechanically ventilated during the intervention period. Arterial blood gases were measured at baseline and serially to guide respiratory rate adjustments to remain within the target Paco_2_ range. A target Richmond Agitation-Sedation Scale score of –4 was achieved by titrating propofol in combination with fentanyl.^16^ Propofol was replaced with midazolam in hemodynamically unstable patients. Neuromuscular blockade was administered if needed. Active cooling with a surface cooling device (Arctic Sun, BD) was initiated immediately after ICU admission with targeted temperature management (TTM) set to 33 °C for the initial 24 hours after cardiac arrest. This was followed by gradual rewarming at 0.5 °C/h and fever control for an additional 48 hours.

For patients allocated to mild hypercapnia, in the event of severe metabolic acidosis (pH < 7.1 and base excess < –6 mM), the initiation of the study protocol commenced after correction of the acidosis. Respiratory acidosis was not corrected with buffer solutions. The return to normocapnia after hypercapnia was conducted over a period of at least 4 hours.

Crystalloid fluids were administered initially in all patients to optimize RV filling pressures with a central venous pressure (CVP) target of 8 to 12 mm Hg. Urine output was targeted at > 1.0 mL/kg/h. Vasopressors and inotropes (primarily norepinephrine and dobutamine, respectively) were used, if needed, to achieve adequate organ perfusion and a target mean arterial pressure of > 65 mm Hg. Emergency coronary angiography, defined as before or immediately after ICU admission, and coronary intervention were performed in all patients demonstrating ST-segment elevation or at the discretion of the treating physician.

### Echocardiographic and Hemodynamic Measurements

We assessed RV function with serial echocardiography performed in conjunction with CVP and RHC when available. RV systolic dysfunction was defined as a minimum of 2 out of 3 measurements of RV systolic function, as defined herein, of less than abnormal thresholds.^17^ RV failure was defined as RV systolic dysfunction, cardiac index of < 2.0 L/min/m^2^, and CVP of > 12 mm Hg.^18^ RV function was evaluated with tricuspid annular plane systolic excursion (TAPSE), pulsed-wave tissue Doppler of the base of lateral free wall (S′), and fractional area change (FAC) of the right ventricle which was calculated as: (end diastolic area – end systolic area / end diastolic area) × 100. Abnormal thresholds were defined as^17^^,^^18^: TAPSE, < 17 mm; S′, < 9.5 cm/s; and FAC, < 35%. Echocardiographic estimation of cardiac index was calculated as: (left ventricular outflow tract velocity time integral × heart rate × left ventricular outflow tract diameter^2^ × 0.7854) / body surface area.

Echocardiography was performed by a cardiologist with a Vivid S70 (General Electric) on admission to the ICU (before the intervention), during intervention, and after the intervention. Examinations were performed in a time-matched fashion with and masked to RHC measurements. For Doppler recordings, the average of at least 3 consecutive beats was calculated. The patient was in a supine position tilted 15° toward the left decubitus position. Because of a limited acoustic window, patient instability, the need for urgent procedures, or a combination thereof, a complete examination was not always possible at ICU admission. Accordingly, the assessment of RV function at ICU admission was limited to TAPSE. Images were stored digitally in Compacs (MediMatic).

Left ventricular volumes and left ventricular ejection fraction were assessed using the Simpson modified rule from the apical 4-chamber view, and left ventricular ejection fraction of < 40% was defined as dysfunction.^19^ Doppler echocardiography assessing mitral inflow with early and late peak transmitral filling velocities were performed in the apical 4-chamber view with the pulsed-wave Doppler sample placed at the tips of mitral leaflets. Mitral annular motion was assessed by pulsed-wave tissue Doppler at the septal and lateral mitral annulus to measure peak early diastolic velocity, with the mean early diastolic velocity reported.

Advanced hemodynamic monitoring was obtained by ultrasound-guided insertion of a 7.5-French triple lumen balloon-tipped catheter into the pulmonary artery (Swan Ganz; Edwards Lifesciences) in eligible patients, regardless of hemodynamic status. Information from RHC was available for treatment decisions that were made at the discretion of the treating physician.

The first RHC measurements were obtained 4 hours after randomization. All measurements were obtained with the patient in a supine position. Systolic pulmonary artery pressure, diastolic pulmonary artery pressure, and mean pulmonary artery pressure (mPAP) were obtained from the distal port of the pulmonary artery catheter. CVP was obtained from the proximal port of the pulmonary artery catheter or from a central line in the superior vena cava. Systemic mean arterial pressure was acquired from a radial arterial line. Cardiac output was obtained through a thermodilution technique with bolus infusions of 10 to 15 mL of cold 5% glucose. The average of 3 measurements with < 10% variance was used. Pulmonary capillary wedge pressure (PCWP) was measured with the catheter balloon inflated in the wedge position and registered at end expiration.

Indexed systemic and pulmonary vascular resistance were calculated as: SVRI (normal range, 1,970-2,390 dynes × s/cm^5^/m^2^) = (mean arterial pressure – CVP) × 80/cardiac index and PVRI (normal range, 255-285 dynes × s/cm^5^/m^2^) = (mPAP – PCWP) × 80/cardiac index, respectively. RV cardiac power output (CPO; normal range, 0.25-0.44 W) and RV stroke work index (SWI; normal range, 8-12 g/m/beat/m^2^) were calculated as: mPAP × cardiac output × 0.0022 and (mPAP – CVP) × (cardiac index × heart rate × 1,000) × 0.0136, respectively.^20^^,^^21^ RV CPO and RV SWI assesses RV workload and contractility.^21^ Pulmonary artery pulsatility index (normal range, > 2.0) was calculated as: (systolic pulmonary artery pressure – diastolic pulmonary artery pressure / CVP).^21^ Pulmonary artery pulsatility index estimates RV pulsatile load and contractile strength and incorporates CVP as a measure of venous congestion.^21^ Pulmonary effective arterial elastance (normal range, 0.7-1.5 mm Hg/mL), a measure of total RV afterload, was calculated as: systolic pulmonary artery pressure / stroke volume.^22^ Systolic pulmonary artery pressure used to calculate TAPSE to systolic pulmonary artery pressure (SPAP) ratio (values of < 0.31 mm/mm Hg are associated with RV pulmonary arterial uncoupling) either was measured directly with RHC or was calculated as 4 × (maximal tricuspid regurgitation velocity)^2^ + invasively measured CVP.^23^ The TAPSE to SPAP ratio is a surrogate marker of RV pulmonary arterial coupling.^24^

Hemodynamic measurements, ventilator settings, arterial and mixed venous blood samples, IV fluids, and urine output were recorded at ICU admission if available and every 4 hours after in a time-matched manner for 48 hours. The doses of relevant administered drugs were time-matched with RHC.

### Statistical Methods

Analyses were performed on an intention-to-treat basis, but were modified by exclusion of patients without echocardiographic assessment. Variables are presented as mean (SD), proportion (%), or median (interquartile range) for nonnormal distributions. Differences between baseline or admission characteristics, coronary pathologic features, and independent echocardiography measurements were compared using the Student *t* test, the χ^2^ test, the Fisher exact test, the Mann-Whitney *U* test, or multivariable linear regression, as appropriate. Tests were 2-sided, and statistical significance was defined as *P* < .05. Nonparametric multivariable imputation by the chained random forest method (missRanger package) was used to replace missing values.^25^

Repeated measurements of echocardiography, RHC, metabolic, respiratory, and intervention variables were evaluated with linear mixed-effects models with a random intercept and slope. All models had treatment group, time, and the interaction term treatment group × time (if significant) as fixed effects and were adjusted for sex and age. Higher-order polynomials of time were used, if needed, to address curvilinearity. Overall differences between treatment groups are reported as estimated marginal means and *P* values, denoted as *P* value for group. Cox proportional hazards models, adjusted for age, sex, time to return of spontaneous circulation, and shockable rhythm, were used for survival analysis. Statistical analysis and graphing were performed with R version 2025.05.1 software (R Foundation for Statistical Computing).

## Results

Altogether 138 patients were randomized, and 111 patients underwent echocardiography, with 52 and 59 patients randomized to hypercapnia and normocapnia, respectively (e-Fig 1). Serial RHC was performed in 84 patients, with 41 patients randomized to hypercapnia and 43 patients randomized to normocapnia. No significant differences in baseline characteristics between patients with or without a pulmonary artery catheter were found (e-Table 1). All patients underwent coronary angiography immediately after admission. Baseline characteristics, coronary angiography findings, doses of sedatives and analgesics, and doses of norepinephrine and dobutamine did not differ significantly between groups (*P* > .05) (Table 1, e-Table 2, e-Fig 2). During the 48 hours of observation, the hypercapnia group received less fluid resuscitation (*P* = .027 for group) (e-Fig 2). Median time from admission to echocardiography during the intervention was longer in the hypercapnia group vs the normocapnia group: 20 hours (interquartile range, 14-23 hours) compared with 15 hours (interquartile range, 5-21 hours; *P* = .003).

**Table 1:** Baseline and Admission Characteristics of Patients Randomized to Targeted Mild Hypercapnia or Targeted Normocapnia After Out-of-Hospital Cardiac Arrest

Characteristic	Hypercapnia (n = 52)	Normocapnia (n = 59)	*P* Value
Age, y	62 (52-67)	64 (57-74)	.063
Female sex	9 (17)	13 (22)	.5
Initial rhythm			.6
Pulseless electrical activity	5 (9.6)	2 (3.4)	
ROSC after bystander defibrillation	3 (5.8)	4 (6.8)	
Ventricular fibrillation	43 (83)	51 (86)	
Ventricular tachycardia	1 (1.9)	2 (3.4)	
Witnessed arrest	46 (88)	53 (90)	.8
Bystander CPR	46 (88)	51 (86)	.7
Time to ROSC, min	20 (15, 35)	23 (15-35)	> .9
Time to advanced life support, min	8.0 (5.0-11.0)	8.5 (5.0-11.0)	.9
Time to needle from arrest, min	100 (70-130)	100 (70-130)	.7
ST elevations on ECG	25 (48)	27 (46)	.8
Known IHD	17 (33)	16 (27)	.5
Previous percutaneous coronary intervention	13 (25)	10 (17)	.3
Previous CABG	8 (15)	7 (12)	.6
Previous myocardial infarction	13 (25)	15 (25)	> .9
Chronic kidney disease	1 (1.9)	0 (0)	.5
Medically treated hypertension	20 (38)	29 (50)	.2
Diabetes mellitus	6 (12)	12 (20)	.2
COPD	4 (7.7)	9 (15)	.2
Peripheral vascular disease	4 (7.7)	4 (6.8)	> .9
Cerebrovascular disease	3 (5.8)	4 (6.8)	> .9
Congestive heart failure	11 (21)	16 (27)	.5
Prior known right ventricular dysfunction	1 (3.4)	2 (7.1)	.6
Assumed cause of cardiac arrest			.4
Acute myocardial infarction	27 (52)	23 (39)	
Hypokalemia	0 (0)	1 (1.7)	
Idiopathic arrhythmia	7 (13)	10 (17)	
Chronic ischemic heart disease	18 (35)	25 (42)	

Data are presented as No. (%) or median (interquartile range) unless otherwise indicated. Differences were compared using the Mann-Whitney *U* test, the χ^2^ test, or the Fisher exact test. Significance level was set to *P* < .05. CABG = coronary artery bypass grafting; IHD = ischemic heart disease; ROSC = return of spontaneous circulation.

### Ventilation and Intervention

**Figure 1:**
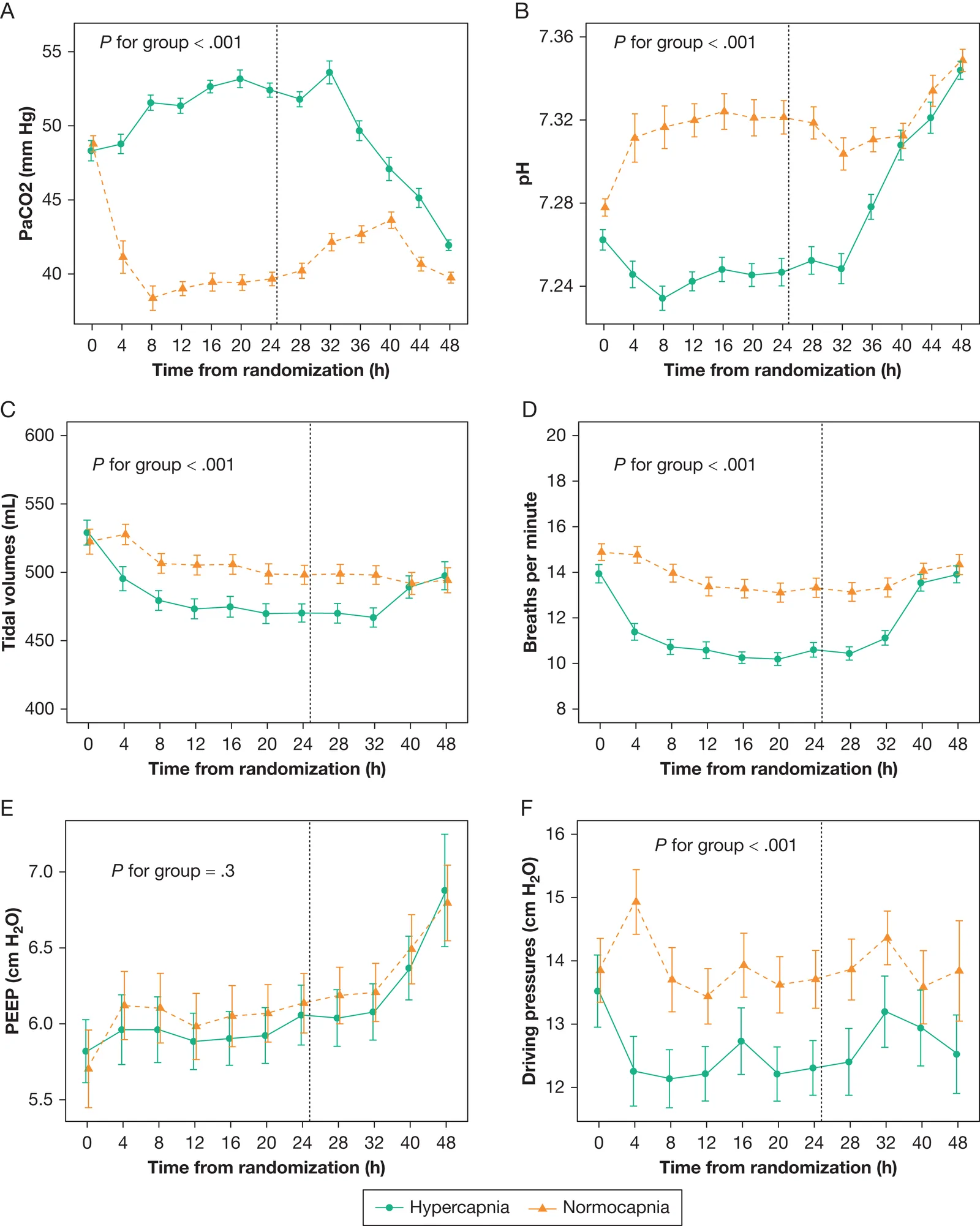
A-F, Graphs showing intervention and ventilation characteristics during targeted mild hypercapnia and targeted normocapnia: arterial partial pressure of CO_2_ (A). pH (B), tidal volumes (C), respiratory rates (D), positive end-expiratory pressure (E), and driving pressures (F). Points represent the mean, and error bars represent the 95% CI. *P* values for group denote the significance of the overall difference between treatment groups, which were analyzed with linear mixed-effects models. The 24-hour intervention period is highlighted with the dotted line. PEEP = positive end-expiratory pressure.

On average, both groups achieved the target values for Paco_2_, and hypercapnia was associated with respiratory acidosis (*P* < .001 for group) (Fig 1). Targeting hypercapnia was associated with reduced tidal volumes and respiratory rates (*P* < .001 for group) (Fig 1). Positive end-expiratory pressure and Pao_2_ to Fio_2_ ratio were similar between groups (*P* > .05) (Fig 1, e-Fig 3).

### Assessment of the Right Ventricle

**Figure 2:**
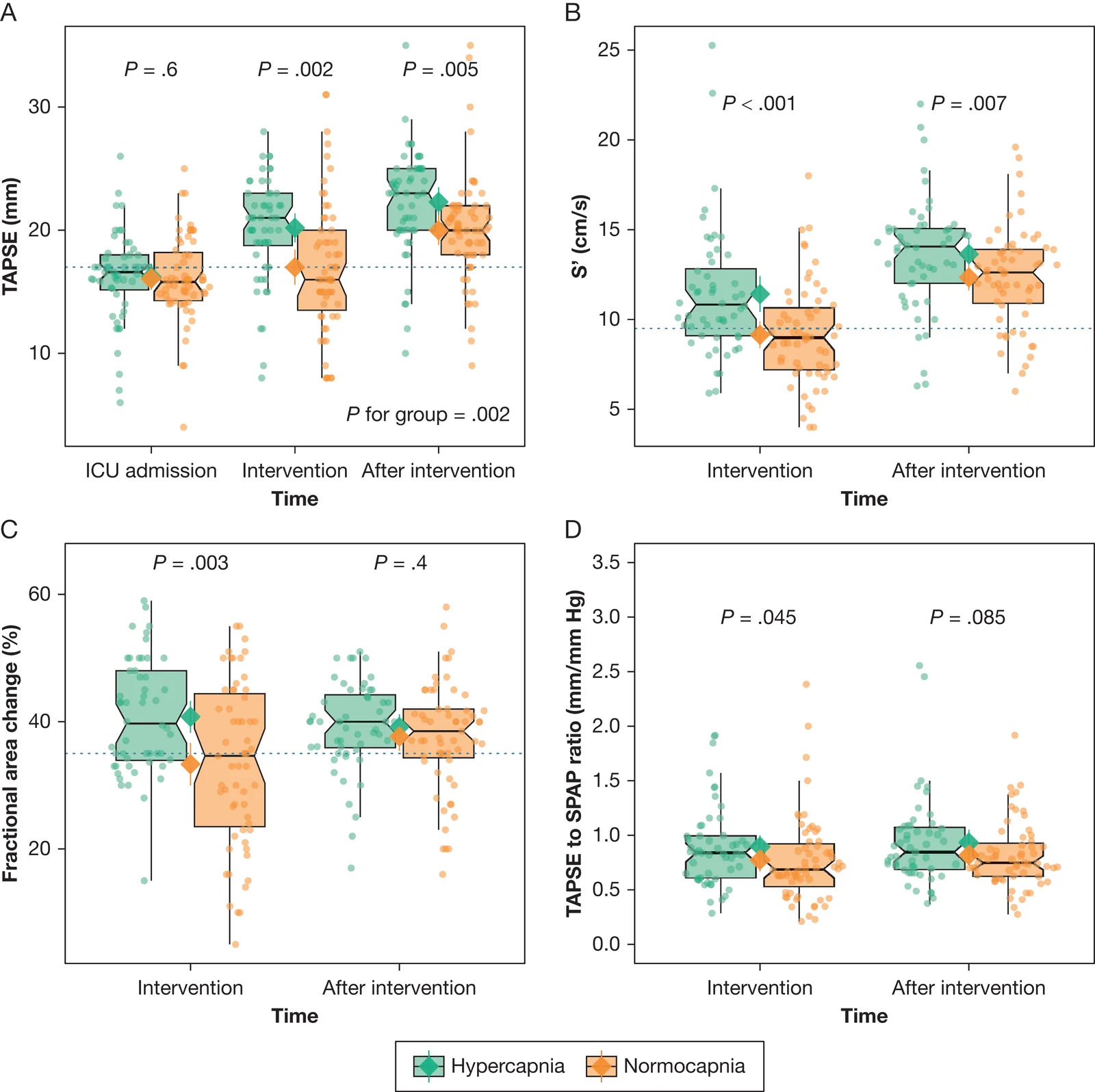
A-D, Boxplots displaying the differences between targeted mild hypercapnia and targeted normocapnia during and after the intervention: TAPSE (A), S′ (B), fractional area change (C), and the ratio of TAPSE to SPAP (D). Differences at single time points were analyzed separately with a Mann-Whitney U test, with significance denoted as a *P* value. Serial TAPSE measurements were assessed with a linear mixed model with significance denoted as *P* value for group. The blue dotted line indicates the abnormal threshold if available for the variable. Diamond-shaped dots represent mean values with 95% CIs. S′ = pulsed-wave tissue Doppler of the base of lateral free wall; SPAP = systolic pulmonary artery pressure; TAPSE = tricuspid annular plane systolic excursion.

At ICU admission, the median TAPSE was approximately 16 mm in both treatment arms (*P* = .3) (Table 2, Fig 2). During the intervention, the median values of TAPSE, S′, and FAC were higher in the hypercapnia group (*P* < .05) (Table 2, Fig 2). To account for the differences in time to first echocardiography, we assessed TAPSE with a mixed-effects model and S′ and FAC with linear regression adjusted for time to first echocardiography. These analyses found similar differences between treatment groups (*P* < .05) (Fig 2, e-Fig 4, e-Appendix 1). Thirteen patients (25%) in the hypercapnia group met the criteria for RV dysfunction vs 37 patients (64%) in the normocapnia group (*P* < .001) (Table 2). After the intervention, TAPSE and S′ were still higher in the hypercapnia group (*P* < .05) (e-Table 3, Fig 2). However, the median values were higher than the normal range in both groups. The TAPSE to SPAP ratio was increased in the hypercapnia group during the intervention (*P* = .045) (Table 2, Fig 2). After the intervention (a median of 42 hours after ICU admission), the criteria for RV systolic dysfunction were met in 10% and 17 % of the patients in the hypercapnia and normocapnia groups, respectively (*P* = .3).

**Table 2:** Echocardiographic and Invasive Hemodynamic Assessment of Right Ventricular Function During Targeted Mild Hypercapnia and Normocapnia

Characteristic	Hypercapnia (n = 52)	Normocapnia (n = 59)	*P* Value
RV dysfunction^a^	13 (25)	37 (64)	< .001
Biventricular dysfunction^b^	9 (21)	24 (46)	.013
RV failure^c^	8 (17)	22 (39)	.011
Invasive hemodynamic parameters time matched with echocardiography			
Cardiac index, mL	2.02 (1.78-2.43)	1.76 (1.64-1.92)	< .001
Stroke volume, mL	71 (67-85)	66 (59-76)	.002
PCWP, mm Hg	13.0 (10.0-14.0)	13.0 (10.0-14.0)	> .9
PAPi	2.38 (1.93-2.85)	2.19 (1.81-2.55)	.14
CVP, mm Hg	10.0 (9.0-11.0)	10.0 (8.0-10.7)	.5
sPAP, mm Hg	42 (37-44)	41 (34-44)	.13
mPAP, mm Hg	26.0 (24.0-27.0)	25.0 (22.0-26.4)	.061
Heart rate, beats/min	53 (47-64)	54 (46-73)	.7
RV CPO, watts	0.23 (0.21-0.26)	0.20 (0.15-0.21)	< .001
RV SWI, g × m/m^2^/beat	7.73 (6.70-9.81)	6.29 (4.87-7.36)	< .001
CVP to PCWP ratio, mm Hg	0.80 (0.71-1.00)	0.75 (0.63-0.88)	.12
Pulmonary effective arterial elastance	0.56 (0.50-0.64)	0.62 (0.54-0.69)	.046
Echocardiographic parameters			
Time from admission to echocardiography, h	20 (14-23)	15 (5-21)	.003
LVEF, %	48 (40-55)	41 (30-53)	.05
E to A ratio	1.40 (1.00-1.95)	1.30 (1.10-1.80)	.4
Mitral valve deceleration time, ms	190 (162-216)	199 (179-228)	.14
E to e′ ratio	9.8 (7.7-12.5)	11.3 (8.0-12.7)	.4
Stroke volume, mL	74 (64-85)	60 (46-76)	< .001
Cardiac index, mL/m^2^	2.09 (1.70-2.35)	1.70 (1.40-2.00)	< .001
Tricuspid regurgitation maximal gradient, mm Hg	25 (20-28)	24 (20-28)	.7
Tricuspid maximal velocity, m/s	2.60 (2.34-2.70)	2.50 (2.31-2.67)	.3
Left atrial volume, mL/m^2^	35 (26-42)	35 (28-41)	> .9
TAPSE at ICU admission, mm	16 (15.0-18.0)	15.8 (14.3-18.4)	.3
TAPSE, mm	21.0 (18.5-23.0)	16.0 (13.0-20.0)	< .001
S′, cm/s	10.83 (9.10-12.95)	9.00 (7.10-10.80)	< .001
Right ventricle end-diastolic basal diameter, mm	40.0 (36.0-45.5)	40.0 (36.0-43.0)	.7
Paradoxical septal movement	3 (5.8)	7 (12)	.3
RVFAC, %	40 (34-48)	35 (23-45)	.003
Inferior vena cava diameter, mm	21.0 (17.0-25.0)	21.0 (18.8-24.0)	.7
TAPSE to SPAP ratio	0.84 (0.61-1.00)	0.69 (0.48-0.94)	.045

Data are presented as No. (%) or median (interquartile range) unless otherwise indicated. Differences were compared using the Student *t* test, the χ^2^ test, or the Fisher exact test and Wilcoxon rank-sum test, as appropriate. Significance level is set to *P* < .05. A = late peak transmitral filling velocity; CPO = cardiac power output; CVP = central venous pressure; E = early peak transmitral filling velocity; e’ = peak early diastolic tissue Doppler velocity; FAC = fractional area change; LVEF = left ventricular ejection fraction; mPAP = mean pulmonary artery pressure; PAPi = pulmonary artery pulsatility index; PCWP = pulmonary capillary wedge pressure; RV = right ventricular; SPAP = systolic pulmonary artery pressure; S′ = pulsed-wave tissue Doppler of the base of lateral free wall; SWI = stroke work index; TAPSE = tricuspid annular plane systolic excursion.

a Defined as 2 of 3 measures of RV systolic function (TAPSE, S′, and FAC) less than abnormal thresholds.

b Defined as RV dysfunction and a left ventricular ejection fraction of < 40 %.

c Defined as RV systolic dysfunction, cardiac index of < 2.0 L/min/m^2^ and CVP of > 12 mm Hg.

**Figure 3:**
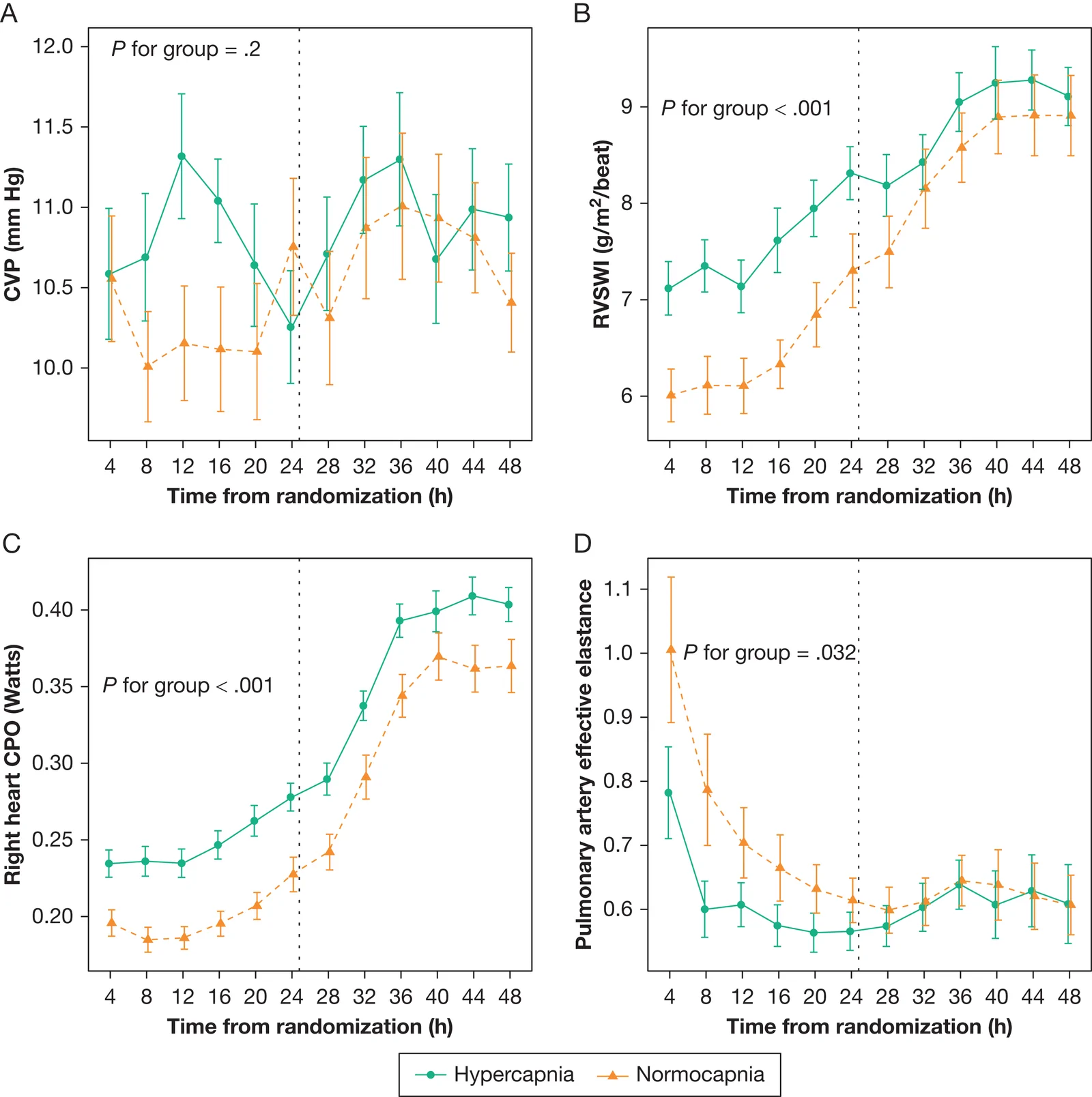
Graphs showing hemodynamic changes during targeted mild hypercapnia and targeted normocapnia: CVP (A), RV SWI (B), right heart CPO (C), and pulmonary arterial effective elastance (D). Points represent the mean, and error bars represent the 95% CI. P value for group denotes the significance of the overall difference between treatment groups, which were analyzed with linear mixed-effects models. The 24-hour intervention period is highlighted with the dotted line. CPO = cardiac power output; CVP = central venous pressure; RV = right ventricular; SWI = stroke work index.

At the initial RHC, most patients in both treatment arms demonstrated precapillary pulmonary hypertension with an average mPAP of > 20 mm Hg, elevated PVRI, and PCWP of < 15 mm Hg, which gradually declined over 48 hours (e-Fig 5). Mild hypercapnia was associated with increased RV SWI and RV CPO during the intervention (*P* < .001 for group) (Table 2, Fig 3). Serial and time-matched mean values of CVP, PVRI, pulmonary artery pulsatility index, and PCWP did not differ significantly between groups (*P* > .05 for group) (Table 2, Fig 3, e-Fig 5). Pulmonary effective arterial elastance was lower in the hypercapnia group (*P* = .032 for group) (Table 2, Fig 3).

Increased cardiac index and stroke volumes in the hypercapnia group during the intervention were demonstrated by both echocardiography and RHC (*P* < .05) (Table 2, e-Fig 5), and SVRI was lower in the hypercapnia group (*P* < .001 for group) (e-Fig 5). Echocardiographic assessment of RV function reflected the RHC measurements because all patients who met the echocardiographic criteria for RV dysfunction demonstrated RV SWI and RV CPO of less than normal thresholds.

### Survival Analysis

**Figure 4:**
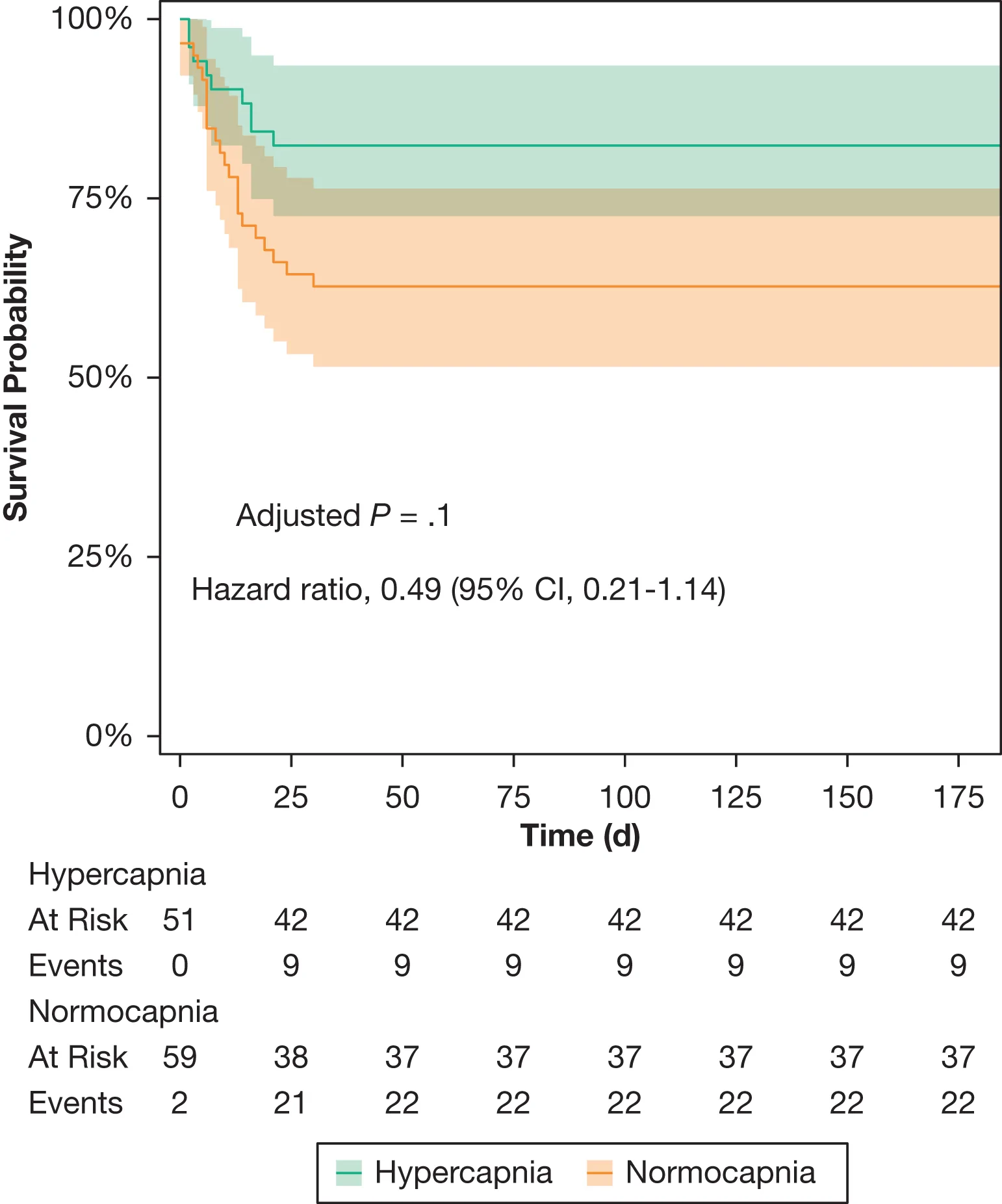
Kaplan-Meier plot displaying the probability of survival at 180 days for patients randomized to targeted mild hypercapnia or normocapnia in the initial 24 hours after randomization. Differences were assessed with a Cox proportional hazard model adjusted for age, sex, shockable rhythm, and time to return of spontaneous circulation. One patient was lost to follow-up.

**Figure 5:**
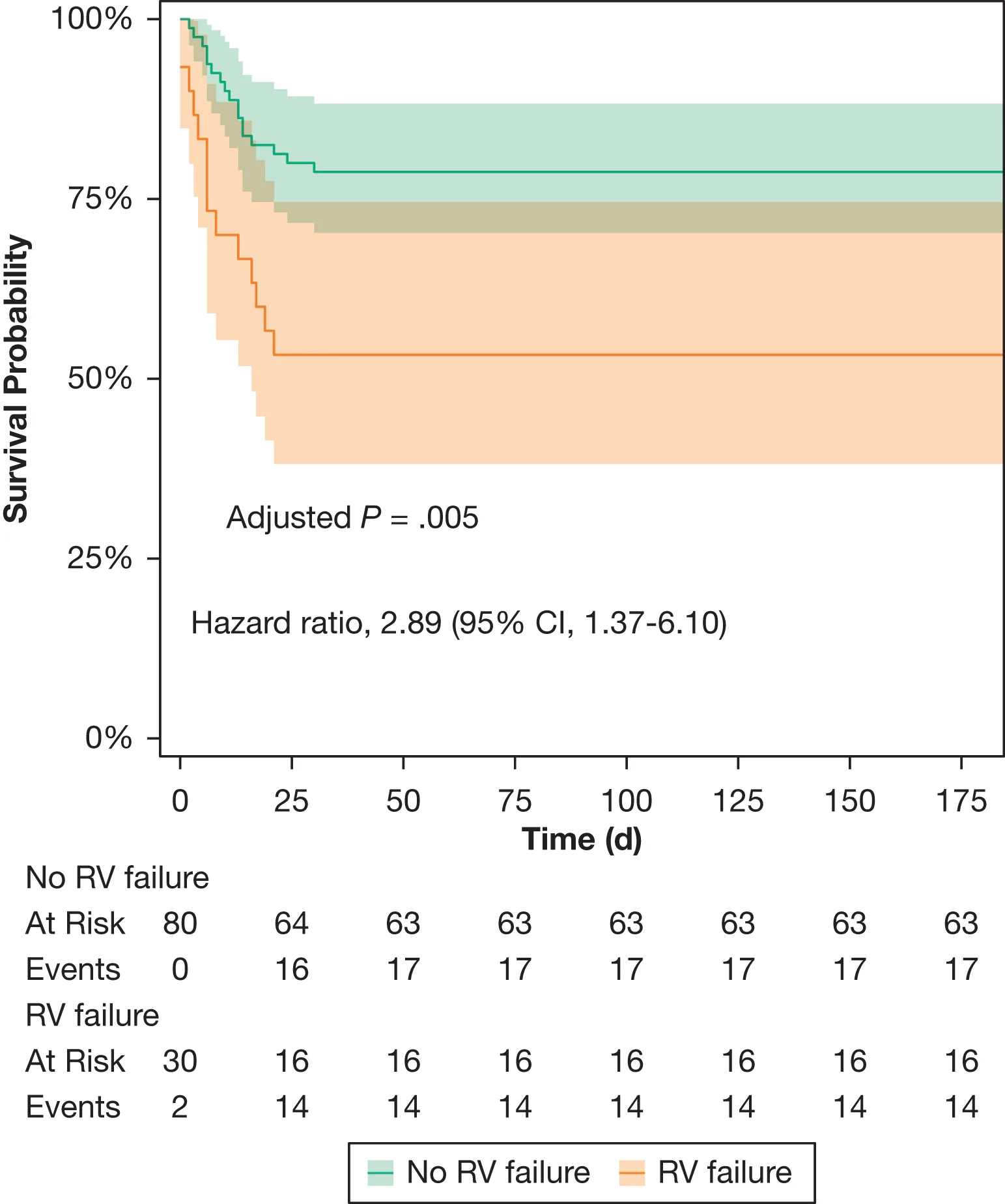
Kaplan-Meier plot displaying the probability of survival with or without RV failure in the initial 48 hours after randomization at 180 days. RV failure was defined as RV systolic dysfunction, cardiac index of < 2.0 L/min/m^2^, and central venous pressure of > 12 mm Hg. Differences were assessed with a Cox proportional hazard model adjusted for age, sex, shockable rhythm, and time to return of spontaneous circulation. One patient was lost to follow-up. RV = right ventricular.

At 6 months, 43 patients (84%) in the hypercapnia group were alive compared with 36 patients (61%) in the normocapnia group; however, this finding was not statistically significant in an adjusted Cox model (*P* = .2) (Table 3, Fig 4). RV failure was associated with an increased 6-month mortality (hazard ratio, 2.89; 95% CI, 1.37-6.10; *P* = .005) (Table 3, Fig 5). Thirty patients (27%) fulfilled the criteria for RV failure, and no significant interaction between treatment group and RV failure was found in the survival analysis (*P* = .6). TAPSE was the only isolated parameter of RV function that remained statistically significant after baseline adjustments: values of < 19 mm were associated with an increased risk of 6-month mortality (hazard ratio, 0.93; 95% CI, 0.86-0.99; *P* = .03) (e-Table 4, e-Fig 6).

**Table 3:** Adjusted Cox Proportional Hazard Models Assessing the Association Between RV Failure Diagnosed in the Initial 48 Hours After Randomization, Treatment Group Allocation, and 6-Month Mortality

Characteristic	HR	95% CI	*P* Value
RV failure	2.89	1.37-6.10	.005
Treatment group (hypercapnia)	0.49	0.21-1.14	.1

Both models were adjusted for the variables age, sex, time to return of spontaneous circulation, and if the initial rhythm was shockable. Of 111 patients randomized, 1 patient was lost to follow-up. HR = hazard ratio; RV = right ventricular.

## Discussion

The main finding of this preplanned substudy of the TAME trial is that targeting mild hypercapnia, compared with normocapnia, for 24 hours in patients resuscitated from OHCA was not associated with worsened RV function. On the contrary, serial assessments using echocardiography and RHC found that mild hypercapnia was associated with significantly improved RV function. Additionally, RV failure within the first 48 hours of care after cardiac arrest was associated with increased 6-month mortality. To our knowledge, this is the first prospective study to report on these associations.

Myocardial dysfunction affecting 1 or both ventricles is an important component of the post-cardiac arrest syndrome, and RV failure is associated with worse outcomes in several critically ill patient populations, such as those with cardiogenic shock, sepsis, and ARDS.^5^^,^^8^, ^9^, ^10^^,^^13^^,^^14^^,^^26^, ^27^, ^28^ A common factor is the need for invasive positive pressure ventilation, in which RV dysfunction and failure presents considerable treatment challenges.^7^ Positive pressure ventilation may reduce RV preload, may increase RV afterload, and may reduce myocardial coronary perfusion.^7^^,^^29^ Consequently, low-to-moderate tidal volume ventilation (6-8 mL/kg) while minimizing positive end-expiratory pressure is recommended for patients with RV failure.^7^^,^^28^^,^^30^ Hypercapnic acidosis, a potential consequence of lowering tidal volumes and minute ventilation, often is discouraged in patients with RV dysfunction because of concerns about increased PVR.^2^^,^^7^^,^^28^^,^^31^ These recommendations are extrapolated from preclinical studies and non-cardiac arrest populations.^7^ The present study provides prospective evidence from patients who are comatose after cardiac arrest, including patients with cardiogenic shock, and directly challenges the prevailing view that mild hypercapnic acidosis and low tidal volumes increase RV afterload and negatively affect RV function.

RV function is influenced by preload, afterload, contractility, lusitropy, pericardial restraint, and interplay with the left ventricle.^18^^,^^32^ Because of the ventilator adjustments and extensive physiologic consequences of hypercapnic acidosis, the precise mechanisms underlying our findings are difficult to isolate.^2^^,^^7^ Diagnosing RV failure at the bedside in the ICU is challenging, and the lack of a consensus definition for RV failure complicates the clinical interpretation of bedside findings.^18^^,^^32^ Pressure volume loops with conductance catheters, which are the gold standard for assessing RV contractility and pulmonary-arterial coupling, were not available in this study.^24^ It is possible that modest reductions in tidal volumes and respiratory rates when targeting hypercapnia improved RV function by favorably altering loading conditions. However, the positive end-expiratory pressure levels, which can impact loading conditions significantly, were similar between the 2 treatment groups.

The only parameter of RV loading that was significantly different between treatment groups was the pulmonary effective arterial elastance, which reflects both resistive and pulsatile elements.^22^ Lower pulmonary effective arterial elastance in the hypercapnia group suggests that targeting hypercapnia reduced RV afterload.^33^ RV stroke work index, RV cardiac power output, and TAPSE to SPAP ratio are associated with RV contractility and collectively suggest improved pulmonary arterial coupling in the hypercapnia group.^24^ Improved RV function may be reflected in the finding that the hypercapnia group received less fluid resuscitation while maintaining similar loading conditions and higher stroke volumes.^13^

This study, in accordance with prior evidence, suggests that a considerable proportion of patients resuscitated from OHCA have myocardial dysfunction in the early phases of care after resuscitation that gradually recovers.^5^^,^^11^ Myocardial stunning resulting from ischemia-reperfusion injury likely plays an important role in this phenomenon, and hypercapnia may influence this recovery positively.^5^^,^^14^^,^^34^, ^35^, ^36^ Hypercapnic acidosis causes systemic and coronary vasodilation, and animal data suggest attenuation of ischemia-reperfusion injury.^2^^,^^4^^,^^34^ Thus, the more rapid recovery of RV function during hypercapnia in this study may be a direct effect of hypercapnic acidosis on myocardial contractility.^4^^,^^34^ Improved LV function secondary to systemic vasodilation likely would benefit RV function and, at least in part, may explain our findings.^15^^,^^24^

In the present study, the definition of RV failure required RV dysfunction on echocardiography with the concurrent inability of the right ventricle to generate sufficient cardiac output and to manage venous return effectively. Accordingly, RV failure was associated with increased 6-month mortality, which strengthens our confidence in the clinical usefulness of such a multimodal assessment. These findings from a single center should be regarded as hypothesis generating and external validity is uncertain, but the findings are consistent with results from other critically ill populations and underscores the importance of identifying and managing RV failure.^10^^,^^26^, ^27^, ^28^ Compared with the parent TAME trial, this substudy found numerically higher survival rates in the hypercapnia group; however, this finding is not statistically significant, and the study was not powered to this assess difference.

### Study Limitations

This study involved a small sample size from a single center. All patients received TTM at 33 °C, and it is unknown whether the results can be reproduced in patients managed with different temperature control strategies. However, RV function was found to be similar between those who received TTM at 33 °C and those who received TTM at 36 °C in a substudy of the TTM trial.^11^ The lack of a universal definition for RV failure complicates generalizability. To mitigate this, we used a standardized, reproducible, multimodal approach. Additionally, our measures of RV function are load dependent, which reduces mechanistic insight. TAPSE was the only consistently measured parameter of RV function at ICU admission, and the median time from randomization to the first echocardiography was longer in the hypercapnia group. Importantly, statistical adjustments for this time difference did not change the results, and RHC suggests that the differences were present throughout the intervention. Only 84 of 111 patients underwent RHC, which limits internal consistency, but we found no significant differences in baseline characteristics between patients with or without RHC.

## Interpretation

Targeting mild hypercapnia for 24 hours in comatose patients with OHCA was not associated with impaired RV function. On the contrary, hypercapnia was associated with improved RV systolic function, decreased systemic vascular resistance, and increased stroke volume. RV failure was associated with increased 6-month mortality, highlighting the need for further research to validate its role as an independent predictor of mortality after OHCA.

## Funding/Support

This study was funded by 10.13039/501100000925National Health and Medical Research Council of Australia, the Health Research Board of Ireland, the Health Research Council of New Zealand, and the 10.13039/501100006095Southern and Eastern Norway Regional Health Authority.

## Financial/Nonfinancial Disclosures

None declared.

## Supplementary Data

e-Online Data

e-Online Data

e-Online Data

e-Online Data

e-Online Data

e-Online Data

e-Online Data

e-Online Data

e-Online Data

Audio
