# Supplementary material for: The Effects of Targeted Mild Hypercapnia on Right Ventricular Function After Out-of-Hospital Cardiac Arrest: A Substudy of the Targeted Therapeutic Mild Hypercapnia After Resuscitated Cardiac Arrest Trial
Source: Chest. 2025 Nov 24;170(1):105–17. doi: 10.1016/j.chest.2025.11.017 (PMC13494458; doi:10.1016/j.chest.2025.11.017)
Supplement: Supplementary file 1 [file mmc5.pdf]

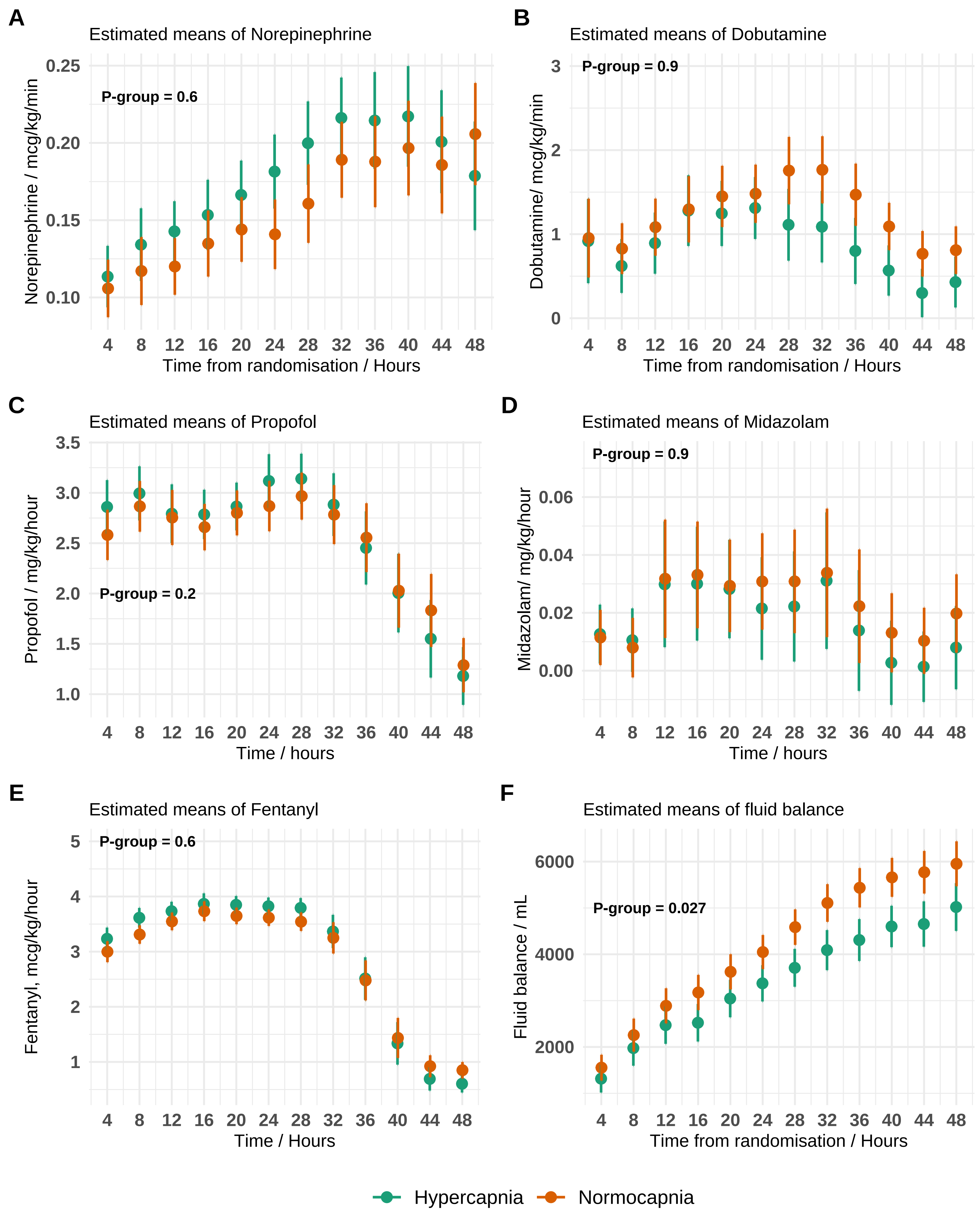
A
Estimated means of Norepinephrine
P-group = 0.6
Norepinephrine / mcg/kg/min
0.25
0.20
0.15
0.10
4 8 12 16 20 24 28 32 36 40 44 48
Time from randomisation / Hours
B
Estimated means of Dobutamine
P-group = 0.9
Dobutamine/ mcg/kg/min
3
2
1
0
4 8 12 16 20 24 28 32 36 40 44 48
Time from randomisation / Hours
C
Estimated means of Propofol
P-group = 0.2
Propofol / mg/kg/hour
3.5
3.0
2.5
2.0
1.5
1.0
4 8 12 16 20 24 28 32 36 40 44 48
Time / hours
D
Estimated means of Midazolam
P-group = 0.9
Midazolam/ mg/kg/hour
0.06
0.04
0.02
0.00
4 8 12 16 20 24 28 32 36 40 44 48
Time / hours
E
Estimated means of Fentanyl
P-group = 0.6
Fentanyl, mcg/kg/hour
5
4
3
2
1
4 8 12 16 20 24 28 32 36 40 44 48
Time / Hours
F
Estimated means of fluid balance
P-group = 0.027
Fluid balance / mL
6000
4000
2000
4 8 12 16 20 24 28 32 36 40 44 48
Time from randomisation / Hours
Hypercapnia
Normocapnia
