# Supplementary material for: The Effects of Targeted Mild Hypercapnia on Right Ventricular Function After Out-of-Hospital Cardiac Arrest: A Substudy of the Targeted Therapeutic Mild Hypercapnia After Resuscitated Cardiac Arrest Trial
Source: Chest. 2025 Nov 24;170(1):105–17. doi: 10.1016/j.chest.2025.11.017 (PMC13494458; doi:10.1016/j.chest.2025.11.017)
Supplement: Supplementary file 2 [file mmc6.pdf]

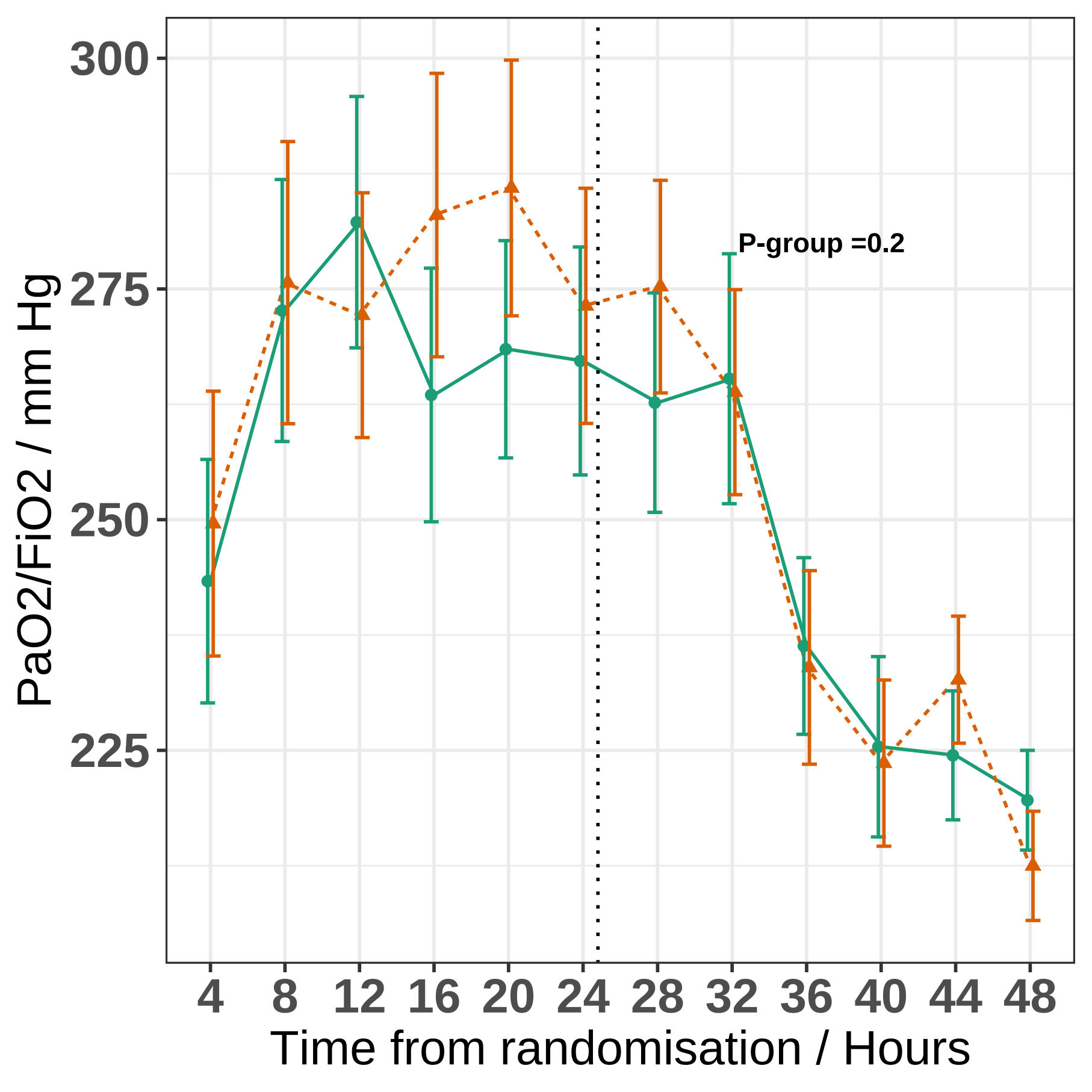

P-group =0.2
300
275
250
225
PaO2/FiO2 / mm Hg
4
8
12
16
20
24
28
32
36
40
44
48
Time from randomisation / Hours
