# Supplementary material for: The Effects of Targeted Mild Hypercapnia on Right Ventricular Function After Out-of-Hospital Cardiac Arrest: A Substudy of the Targeted Therapeutic Mild Hypercapnia After Resuscitated Cardiac Arrest Trial
Source: Chest. 2025 Nov 24;170(1):105–17. doi: 10.1016/j.chest.2025.11.017 (PMC13494458; doi:10.1016/j.chest.2025.11.017)
Supplement: Supplementary file 3 [file mmc7.pdf]

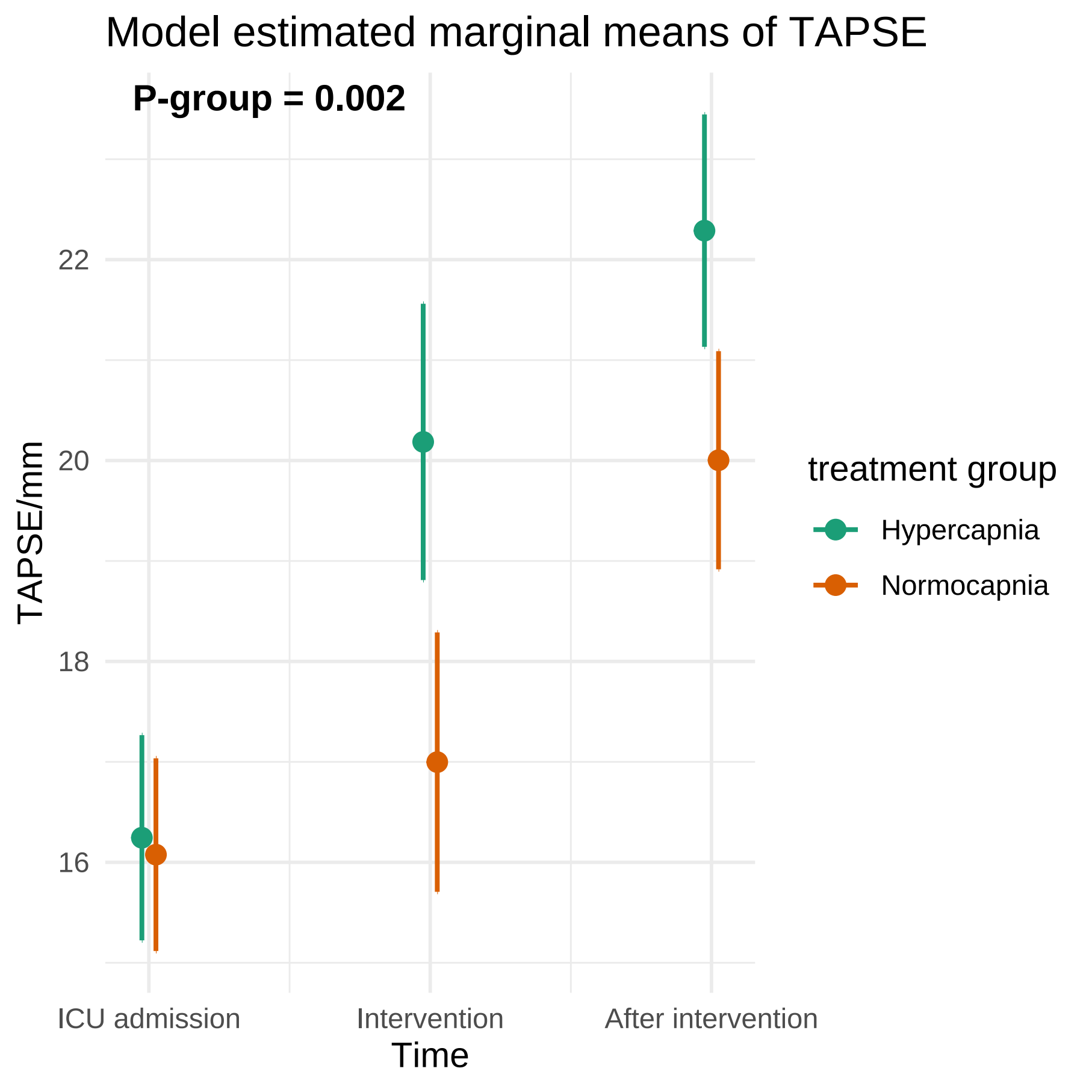
Model estimated marginal means of TAPSE
P-group = 0.002
TAPSE/mm
16
18
20
22
ICU admission
Intervention
After intervention
Time
treatment group
Hypercapnia
Normocapnia
