# Supplementary material for: The Effects of Targeted Mild Hypercapnia on Right Ventricular Function After Out-of-Hospital Cardiac Arrest: A Substudy of the Targeted Therapeutic Mild Hypercapnia After Resuscitated Cardiac Arrest Trial
Source: Chest. 2025 Nov 24;170(1):105–17. doi: 10.1016/j.chest.2025.11.017 (PMC13494458; doi:10.1016/j.chest.2025.11.017)
Supplement: Supplementary file 4 [file mmc8.pdf]

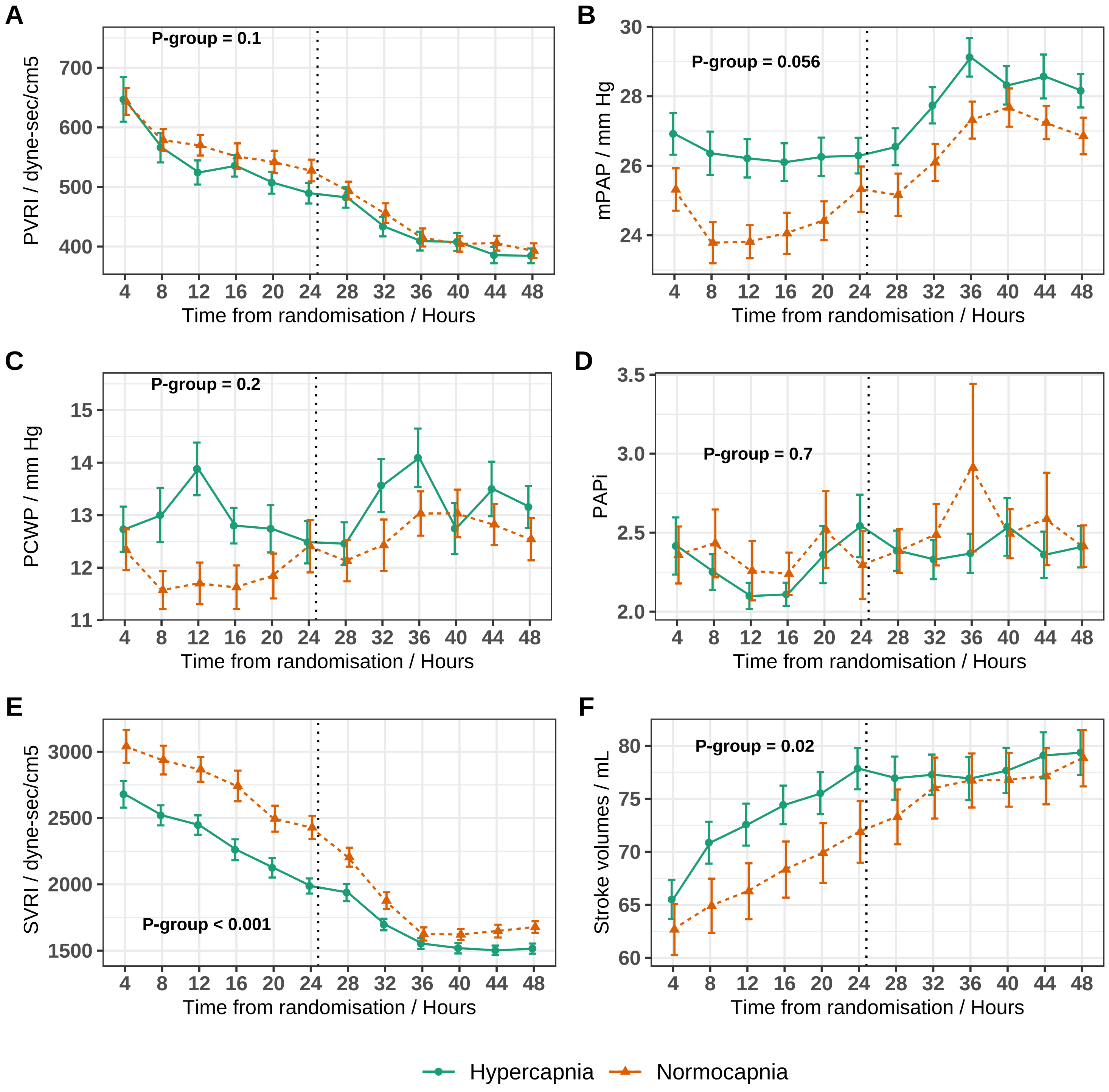
A
P-group = 0.1
PVRI / dyne-sec/cm5
700
600
500
400
4 8 12 16 20 24 28 32 36 40 44 48
Time from randomisation / Hours
B
P-group = 0.056
mPAP / mm Hg
30
28
26
24
4 8 12 16 20 24 28 32 36 40 44 48
Time from randomisation / Hours
C
P-group = 0.2
PCWP / mm Hg
15
14
13
12
11
4 8 12 16 20 24 28 32 36 40 44 48
Time from randomisation / Hours
D
P-group = 0.7
PAPi
3.5
3.0
2.5
2.0
4 8 12 16 20 24 28 32 36 40 44 48
Time from randomisation / Hours
E
P-group < 0.001
SVRI / dyne-sec/cm5
3000
2500
2000
1500
4 8 12 16 20 24 28 32 36 40 44 48
Time from randomisation / Hours
F
P-group = 0.02
Stroke volumes / mL
80
75
70
65
60
4 8 12 16 20 24 28 32 36 40 44 48
Time from randomisation / Hours
Hypercapnia
Normocapnia
