# Supplementary material for: The Effects of Targeted Mild Hypercapnia on Right Ventricular Function After Out-of-Hospital Cardiac Arrest: A Substudy of the Targeted Therapeutic Mild Hypercapnia After Resuscitated Cardiac Arrest Trial
Source: Chest. 2025 Nov 24;170(1):105–17. doi: 10.1016/j.chest.2025.11.017 (PMC13494458; doi:10.1016/j.chest.2025.11.017)
Supplement: Supplementary file 6 [file mmc1.docx]

**Electronic supplementary Material**

Results of the assessment of echocardiographic measurements of right ventricular function during targeted mild hypercapnia or normocapnia. This was assessed with robust linear regression adjusted for time from randomization to first echocardiography.

**Assessment of S’:**

**Assessment of fractional area change (FAC):**

**Assessment of TAPSE: S** **PAP – ratio:
Including both adjusted and un-adjusted model output.**
