# Supplementary material for: The Effects of Targeted Mild Hypercapnia on Right Ventricular Function After Out-of-Hospital Cardiac Arrest: A Substudy of the Targeted Therapeutic Mild Hypercapnia After Resuscitated Cardiac Arrest Trial
Source: Chest. 2025 Nov 24;170(1):105–17. doi: 10.1016/j.chest.2025.11.017 (PMC13494458; doi:10.1016/j.chest.2025.11.017)
Supplement: Supplementary file 7 [file mmc2.docx]

**Electronic supplementary figure legends:**

**E- Figure 2:** Differences in relevant drugs and fluid administration during targeted mild hypercapnia and targeted normocapnia. A, Norepinephrine. B, Dobutamine C, Propofol. D, Midazolam. E, Fentanyl. F, Positive fluid balance.

Points represent the estimated mean from the mixed effect model, and error bars represent a 95% confidence interval. *P*-group denotes the significance of the overall difference between treatment groups, which were analyzed with linear mixed effects models. The 24-hour intervention period is highlighted with the dotted line.

**E-Figure 3**: Differences in the partial pressure of oxygen to inspired fraction of oxygen -ratio (PaO2 / FiO2 -ratio) during targeted mild hypercapnia and normocapnia.

Points represent the estimated mean from the mixed effect model, and error bars represent a 95% confidence interval. *P*-group denotes the significance of the overall difference between treatment groups, which were analyzed with linear mixed effects models. The 24-hour intervention period is highlighted with the dotted line.

**E- Figure 4:** Estimated marginal means from a linear mixed effect model displaying the differences in tricuspid annular plane systolic excursion during targeted mild hypercapnia and normocapnia. The model was adjusted for the time from randomization to echocardiography, age and sex. P-group denotes the significance of the overall difference between treatment groups.

**E- Figure 5:** Differences in invasive hemodynamics during targeted mild hypercapnia and targeted normocapnia. A, Pulmonary vascular resistance. B, Mean pulmonary artery pressure. C, Pulmonary capillary wedge pressure. D, Pulmonary artery pulsatility index. E, Systemic vascular resistance index. F, Stroke volume.

Points represent the estimated mean from the mixed effect model, and error bars represent a 95% confidence interval. *P*-group denotes the significance of the overall difference between treatment groups, which were analyzed with linear mixed effects models. The 24-hour intervention period is highlighted with the dotted line.

**E-Figure 6:** Graphical depiction of the association between tricuspid annular plane systolic excursion (TAPSE) sampled during initial 24 hours after out-of-hospital cardiac arrest and 6-month mortality. This was assessed with a Cox proportional hazard model adjusted for age, sex and time to ROSC. The figure displays the hazard ratios for different TAPSE levels with 95 % confidence intervals, relative to the median TAPSE-value. The horizontal, black line illustrates the hazard ratio = 1. The vertical, red line marks the median TAPSE value at 19 mm.
