# Supplementary material for: The Effects of Targeted Mild Hypercapnia on Right Ventricular Function After Out-of-Hospital Cardiac Arrest: A Substudy of the Targeted Therapeutic Mild Hypercapnia After Resuscitated Cardiac Arrest Trial
Source: Chest. 2025 Nov 24;170(1):105–17. doi: 10.1016/j.chest.2025.11.017 (PMC13494458; doi:10.1016/j.chest.2025.11.017)
Supplement: Supplementary file 8 [file mmc3.docx]

**Supplementary E-Figure 1**

**CONSORT Flow Diagram**

**Excluded (n= 281)**

**Inclusion not met (n= 250)**

- Non-cardiac cause (n= 128)

- Sustained ROSC > 20 minutes (n= 20)

- FOUR-score motor response > 4 (n= 73)

- Eligible for ICU without restriction (n= 21)

- Within 180 minutes (n= 8)

**Exclusion met (n= 31)**

- Unwitnessed and asystole (n= 9)

- ECMO (n=22)

## Enrollment

Randomised (n= 139)

**Eligible, not randomized (n= 19)**
- Transfer (n=7)
- Mental incapacity (n=1)
- Clinician decision (n=11)

Assessed for eligibility (n = 439)

Allocated to TN (n = 71)

- No consent (n = 1)

- Echocardiography not performed or not stored for analysis (n = 12)

Allocated to TMH (n = 68)

- No consent (n =1)

- Echocardiography not performed or not stored for analysis (n = 16)

## Allocation

## Included in the present sub-study and assessed with echocardiography.

TMH (n = 52)

TN (n = 59)

- Assessed with right heart catheterization (n=43).

- Assessed with right heart catheterization (n=41).

Consort diagram depicting the flow of patients included in the present sub-study of the TAME trial at Oslo University Hospital. TMH indicates targeted mild hypercapnia; TN, targeted normocapnia; hs-cTnT
