# Supplementary material for: The Effects of Targeted Mild Hypercapnia on Right Ventricular Function After Out-of-Hospital Cardiac Arrest: A Substudy of the Targeted Therapeutic Mild Hypercapnia After Resuscitated Cardiac Arrest Trial
Source: Chest. 2025 Nov 24;170(1):105–17. doi: 10.1016/j.chest.2025.11.017 (PMC13494458; doi:10.1016/j.chest.2025.11.017)
Supplement: Supplementary file 9 [file mmc4.docx]

**SUPPLEMENTARY TABLES**

**E-Table 1) Baseline characteristics for patients with or without a pulmonary artery catheter.**

| **Characteristic** | **Pulmonary artery catheter** N = 84^1^ | **No pulmonary artery catheter** N = 27^1^ | **p-value**^2^ |
| --- | --- | --- | --- |
| Age | 62 (54, 69) | 66 (58, 72) | 0.3 |
| Sex |  |  | 0.5 |
| Female | 14 (18%) | 8 (24%) |  |
| Male | 63 (82%) | 26 (76%) |  |
| Treatment group |  |  | 0.7 |
| Hypercapnia | 37 (48%) | 15 (44%) |  |
| Normocapnia | 40 (52%) | 19 (56%) |  |
| Initial rhythm |  |  | 0.3 |
| Pulseless electric activity | 3 (3.9%) | 4 (12%) |  |
| ROSC after bystander defibrillation | 4 (5.2%) | 3 (8.8%) |  |
| Ventricular fibrillation | 68 (88%) | 26 (76%) |  |
| Ventricular tachycardia | 2 (2.6%) | 1 (2.9%) |  |
| Witnessed arrest | 70 (91%) | 29 (85%) | 0.5 |
| Bystander CPR | 68 (88%) | 29 (85%) | 0.8 |
| Time to ROSC | 20 (14, 35) | 23 (15, 40) | 0.3 |
| Time to advanced life support | 8.0 (4.0, 11.0) | 10.0 (5.0, 10.0) | 0.8 |
| ST-elevation myocardial infarction | 38 (49%) | 14 (41%) | 0.4 |
| Known ischemic heart disease | 24 (31%) | 9 (26%) | 0.6 |
| Previous percutaneous coronary intervention | 17 (22%) | 6 (18%) | 0.6 |
| Previous coronary bypass grafting | 11 (14%) | 4 (12%) | >0.9 |
| Previous myocardial infarction | 20 (26%) | 8 (24%) | 0.8 |
| Previously known renal disease | 0 (0%) | 1 (2.9%) | 0.3 |
| Previously treated hypertension | 34 (44%) | 15 (45%) | >0.9 |
| Diabetes mellitus | 12 (16%) | 6 (18%) | 0.8 |
| Chronic obstructive pulmonary disease | 8 (10%) | 5 (15%) | 0.5 |
| Peripheral vascular disease | 7 (9.1%) | 1 (2.9%) | 0.4 |
| Cerebrovascular disease | 5 (6.5%) | 2 (5.9%) | >0.9 |
| Congestive heart failure | 21 (27%) | 6 (18%) | 0.3 |
| Prior right ventricular dysfunction | 1 (2.6%) | 2 (11%) | 0.3 |
| Assumed cause of arrest |  |  | 0.6 |
| AMI | 34 (44%) | 16 (47%) |  |
| Hypokalemia | 0 (0%) | 1 (2.9%) |  |
| Idiopathic arrhythmia | 13 (17%) | 4 (12%) |  |
| Chronic ischemic origin | 29 (38%) | 13 (38%) |  |
| Unknown | 1 (1.3%) | 0 (0%) |  |
| ^1^Median (Q1, Q3); n (%) | | | |
| ^2^Wilcoxon rank sum test; Pearson's Chi-squared test; Fisher's exact test | | | |

**E-Table 2: *Differences in coronary angiography results and coronary intervention for patients randomized to targeted mild hypercapnia or normocapnia.***

| Characteristic | Hypercapnia  N = 52^1^ | Normocapnia  N = 59^1^ | p-value^2^ |
| --- | --- | --- | --- |
| Coronary vessel disease |  |  | >0.9 |
| 1 | 19 (37%) | 24 (41%) |  |
| 2 | 7 (14%) | 7 (12%) |  |
| 3 | 13 (25%) | 15 (25%) |  |
| Atheromatosis without significant stenosis | 7 (14%) | 8 (14%) |  |
| Muscle-bridge | 1 (2.0%) | 0 (0%) |  |
| No coronary pathology | 5 (9.8%) | 5 (8.5%) |  |
| Infarct related artery in patients with AMI |  |  | 0.6 |
| Left main | 1 (1.9%) | 4 (6.8%) |  |
| LAD | 13 (25%) | 11 (19%) |  |
| RCA | 7 (13%) | 11 (19%) |  |
| LCX | 7 (13%) | 5 (8.5%) |  |
| PCI successful |  |  | 0.13 |
| Failed PCI attempt | 5 (13%) | 6 (11%) |  |
| Clinical decision not to intervene | 8 (21%) | 20 (38%) |  |
| Yes | 26 (67%) | 26 (50%) |  |
| Time to needle from arrest, min | 100 (70, 130) | 100 (70, 130) | 0.7 |
| ^1^n (%); Median (Q1, Q3) | | | |
| ^2^Fisher's exact test; Wilcoxon rank sum test; Pearson's Chi-squared test | | | |

Data are presented as proportions or median and lower to upper quartile (Q1–Q3). Differences were compared using Mann U Whitney test, χ2 test or Fisher’s exact test. Significance level is set to p < 0.05. AMI denotes acute myocardial infarction; LAD, left anterior descending artery; RCA, right coronary artery;LCX, left circumflex artery; PCI, percutaneous coronary intervention.

**E-Table 3: *Echocardiographic and invasive hemodynamic assessment of right ventricular function after the intervention with targeted mild hypercapnia and targeted normocapnia.***

| **Characteristic** | **Hypercapnia**  N = 52^1^ | **Normocapnia**  N = 59^1^ | **p-value**^2^ |
| --- | --- | --- | --- |
| **Time matched invasive hemodynamic parameters** | | | |
| CI, mL/m2 | 3.14 (2.81, 3.50) | 2.81 (2.60, 3.07) | 0.008 |
| Stroke volume, mL | 79 (64, 89) | 69 (58, 85) | 0.064 |
| PCWP, mm Hg | 13.3 (11.0, 16.0) | 14.5 (11.1, 16.2) | 0.3 |
| PAPi | 2.43 (1.82, 2.70) | 2.37 (1.83, 2.83) | 0.9 |
| **Echocardiographic parameters** | | | |
| Time from admission to echocardiography, hours | 42 (39, 45) | 42 (37, 46) | 0.7 |
| LVEF, % | 48 (43, 55) | 44 (40, 55) | 0.2 |
| E/A ratio | 1.23 (0.96, 1.50) | 1.20 (1.00, 1.50) | >0.9 |
| Mitral valve deceleration time, ms | 189 (150, 207) | 193 (169, 207) | 0.4 |
| E/e’ | 9.49 (7.70, 10.60) | 10.10 (8.00, 10.60) | 0.7 |
| Stroke volume, mL | 76 (66, 89) | 67 (60, 81) | 0.035 |
| Cardiac index, mL/m2 | 2.84 (2.42, 3.36) | 2.50 (2.31, 3.00) | 0.010 |
| Tricuspid regurgitation maximal gradient, mmHg | 26 (22, 30) | 26 (22, 30) | >0.9 |
| Tricuspid peak systolic velocity, m/s | 2.86 (2.50, 3.17) | 2.90 (2.60, 3.18) | 0.5 |
| Left atrial volume, mL/m2 | 35 (28, 41) | 34 (29, 38) | 0.5 |
| TAPSE, mm | 23.0 (20.0, 25.0) | 20.0 (18.0, 22.0) | <0.001 |
| S’, cm/s | 14.1 (12.0, 15.1) | 12.6 (10.8, 13.9) | 0.007 |
| RV end-diastolic basal diameter, cm | 39.1 (36.5, 41.0) | 40.0 (37.0, 43.0) | 0.2 |
| Paradoxical septal movement, n | 2 (3.8%) | 3 (5.1%) | >0.9 |
| FAC, % | 40 (36, 44) | 39 (34, 42) | 0.4 |
| IVC diameter 2, mm | 21.0 (18.0, 23.7) | 20.8 (20.0, 23.0) | >0.9 |
| TAPSE:SPAP ratio | 0.87 (0.69, 1.09) | 0.75 (0.62, 0.95) | 0.087 |
| ^1^Median (Q1, Q3); n (%) | | | |
| ^2^Wilcoxon rank sum test; Fisher's exact test | | | |

Data are presented as proportions (%) and as median and lower to upper quartile (Q1–Q3). Differences were compared using Student t test, χ2 test, Fisher’s exact test and Wilcoxon rank-sum test, as appropriate. Significance level is set to p-value < 0.05.

PCWP denotes pulmonary capillary wedge pressure; PAPi, pulmonary artery pulsatility index; sPAP, systolic pulmonary artery pressure; mPAP, mean pulmonary artery pressure; CPO, cardiac power output, RVSWI, right ventricular stroke work index; CVP, central venous pressure; LVEF, left ventricular ejection fraction; TAPSE, tricuspid annular plane systolic excursion; S’, pulsed wave tissue Doppler of the base of lateral free wall; TAPSE:SPAP, the ratio of TAPSE to the pulmonary artery systolic pressure.

**E-Table 4) The association between echocardiographic measurements and the probability of death at six-months assessed with unadjusted cox proportional hazard modeling.**

| **Characteristic** | ***HR****^1^* | **95% CI***^1^* | **p-value** |
| --- | --- | --- | --- |
| TAPSE during intervention | 0.91 | 0.85, 0.98 | 0.008 |
| TAPSE after intervention | 0.94 | 0.87, 1.01 | 0.10 |
| S’ during intervention | 0.94 | 0.83, 1.05 | 0.30 |
| S’ after intervention | 0.98 | 0.88, 1.10 | 0.9 |
| RV fractional area change during intervention | 0.97 | 0.94, 1.00 | 0.031 |
| RV fractional area change  after intervention | 0.97 | 0.93, 1.01 | 0.2 |
| Left ventricular ejection fraction during intervention | 0.98 | 0.95, 1.02 | 0.063 |
| Left ventricular ejection fraction after intervention | 0.99 | 0.96, 1.02 | 0.4 |
| Stroke volumes during intervention | 0.97 | 0.95, 0.98 | <0.001 |
| Stroke volumes after intervention | 0.98 | 0.96, 1.00 | 0.027 |
| TAPSE:SPAP ratio during intervention | 0.39 | 0.12, 1.27 | 0.12 |
| Cardiac index during intervention | 0.61 | 0.30, 1.26 | 0.2 |
| Cardiac index after intervention | 0.67 | 0.39, 1.13 | 0.13 |

**Abbreviations: CI = Confidence Interval, HR = Hazard Ratio**
